# Targeting the glycoproteome

**DOI:** 10.1007/s10719-012-9438-6

**Published:** 2012-08-11

**Authors:** Jonas Nilsson, Adnan Halim, Ammi Grahn, Göran Larson

**Affiliations:** Department of Clinical Chemistry and Transfusion Medicine, Institute of Biomedicine, The Sahlgrenska Academy at the University of Gothenburg, Sahlgrenska University Hospital, Gothenburg, 413 45 Sweden

**Keywords:** Glycoproteomics, Glycopeptide, Attachment sites, Liquid chromatography, Tandem mass spectrometry, Enrichment, Lectin affinity, Hydrazide chemistry

## Abstract

Despite numerous original publications describing the structural complexity of N- and O-linked glycans on glycoproteins, only very few answer the basic question of which particular glycans are linked to which amino acid residues along the polypeptide chain. Such structural information is of fundamental importance for understanding the biological roles of complex glycosylations as well as deciphering their non-template driven biosynthesis. This review focuses on presenting and commenting on recent strategies, specifically aimed at identifying the glycoproteome of cultured cells and biological samples, using targeted and global enrichment procedures and utilizing the high resolution power, high through-put capacity and complementary fragmentation techniques of tandem mass spectrometry. The goal is to give an update of this emerging field of protein and glyco-sciences and suggest routes to bridge the data gap between the two aspects of glycoprotein characteristics, *i.e.* glycan structures and their attachment sites.

## Introduction

The four essential building blocks of all cells are the oligonucleotides, proteins, lipids and carbohydrates (glycans). Oligonucleotides and proteins are linear polymers that have only one basic type of linkage between their different building blocks (nucleic and amino acids). In contrast glycans are built from monosaccharide residues that can be linked to each other in linear or branched sequences *via* either α- or β-glycosidic linkages to one of several positions onto a neighbouring monosaccharide [1]. Glycosylation of proteins is one of many post-translational modifications (PTMs) that determine the processing, distribution and metabolism as well as the biological functions of most proteins. The functions of glycans on protein are to ensure correct folding, provide protease resistance and solubility, and to serve as biological ligands for carbohydrate binding proteins. Specific glycans play different roles in growth, development and differentiation, cell-cell interactions, cell migrations, host-microbe interactions and in blood haemostasis [1–4].

Glycosylation is, in contrast to the biosynthesis of DNA, RNA and proteins, understood as a non-template driven enzymatic process that allows for glycan attachment, trimming, chain elongation and branching as well as glycan derivatization. It has been estimated that 1–2 % of the human genome codes for proteins involved in the glycosylation process [5]. Epimerization, deacetylation and sulphation are typical steps for the processing of proteoglycans, a large and important group of glycoproteins whose structural elucidations will not be the subject of this review (see instead [6]). Another group of glycoproteins that will not be covered in this review are the GPI-linked proteins for which we instead refer to a recent review [7].

For most soluble or membrane bound glycoproteins, including mucins and glycopeptides, there are in principal two major forms of glycosylation, which differ principally in their subcellular compartmentalization and processing and which we commonly refer to as N- and O-linked glycosylations. The N- refers to the glycosidic linkage anchoring the glycan to the amide group side chain of the Asn residues, and the O- refers to the glycosidic linkage anchoring the glycan to the gamma hydroxyl groups of Ser and Thr residues of the peptide stretch. The N-linked glycosylation takes place in the endoplasmic reticulum (ER), is catalysed by one oligosaccharyltransferase complex which transfers a preassembled oligosaccharide from its dolichol-anchor to the growing polypeptide chain, and is strictly dependent on the presence of an Asn-X-Ser/Thr/Cys consensus sequence, where X is any amino acid except proline [8, 9]. The GalNAc O-glycosylation of proteins, commonly referred to as the mucin-type O-glycosylation, is initiated by a family of 20 polypeptide *N*-acetylgalactosaminyltransferase (ppGalNAcT) enzymes [10, 11] and is usually found in regions rich in Ser, Thr and Pro residues. Presently, there is no known consensus sequence for mucin-type O-glycosylation, which most likely is due to the different substrate specificities of the various ppGalNAcTs. The details of N- and O-linked glycan biosynthesis are beyond the scope of this review and the readers are instead referred to the following reviews [12–16]. More recently additional unique protein glycosylations have been defined, which play significant roles in signalling, either between cells or between subcellular compartments. The O-fucosylation of Notch [17] and the O-GlcNAc glycosylation of intranuclear proteins [18] are such examples of non-classical glycosylation of proteins that are deeply involved in differentiation and cell signalling [19].

Due to the many theoretical possibilities of linking monosaccharides to each other into branched and linear sequences decades of large efforts have been put into developing—and broadly applying—sensitive and accurate methods for the detailed structural characterization of thousands of glycoconjugates. No single method is however available [20] for a complete characterization of biological glycoconjugates and thus for each and every one of them to be completely assigned in all structural details several chromatographic, mass spectrometric, enzymatic or affinity based as well as physico-chemical methods (*e.g.* NMR spectroscopy) are needed. This is to unambiguously define the constituent monosaccharides, their conformation, linkage positions and configurations, sequence and possible branching, total size and derivatizations. However, the number of theoretical permutations of glycan structures are in reality restricted by the number and types of genes and their corresponding proteins critical for the biosynthesis of the actual glycoconjugates expressed at any given time in a defined cell and organism. Thus, with data from the many glycan structures already defined in detail during the last decades and by the conceptual understanding of genetic restriction, the structural variability is in reality becoming within reach of novel high-throughput analytical methods such as LC-MS/MS. Thus, in many recent publications glycan structures are deduced or postulated from earlier studies of identical or similar material. However, whenever a new set of glycoconjugate structures are identified the tedious work of a complete structural characterization should be encouraged to avoid future confusions. This structural complexity will put pressure and responsibility onto the glyco-science community to keep relevant methods available and updated as well as to include only accurate data into relevant databases.

As much as the complexity of glycoconjugates is an attractive challenge to the glyco-science field it must also be realized as a potential repellent barrier for many other fields of natural sciences. Traditionally, glyco-scientists characterize glycoproteins through a “glycomics” approach cleaving off the glycans, and characterizing them in large detail, but put less effort on the proteins while other scientist interested in the same glycoproteins often cleave off the glycans but concentrate only on the proteins. This often leads to an unnecessary splitting of the structural data. With the advent of global and targeted high-throughput analyses of proteins—and more recently of glycoproteins—we foresee a future possibility for collecting all structural data in the same databases.

In this review of glycoproteomics we will focus on presenting various means of isolating and characterizing glycoproteins from biological sources, to yield reliable and storable data both as to the glycan structures, their attachment sites as well as to protein sequences and identities.

## Lectin and antibody affinity purification for targeted glycoproteomics; *i.e.* single glycoprotein analysis

Glycosylation of proteins offers both advantages and disadvantages when it comes to purification procedures (Table 1). One advantage is that glycans may be used as additional ligands for affinity purification using Carbohydrate Binding Proteins (CBP). However, as glycoproteins may be present as different glycoforms of the same core protein the use of glycan structures for purification may spuriously isolate non representative monospecies glycoproteins. Also, glycans may shield antigenic epitopes and thus it may be more difficult to use antibodies reactive towards the corresponding “core” proteins.

**Table 1:** Common strategies and typical characteristics for enrichment and mass spectrometric analysis of glycoproteins and glycopeptides

	Substrate	Method	Pros/Cons		References
Enrichment					
Targeted	Glycoproteins	Antibody	+	High specificity for targeted glycoproteins	[57, 59, 62, 63, 67]
			−	Limited by availability of specific antibodies	
Global	Glycoproteins	Lectin	+	Readily available and cheap	[72]
			+	Enrichment of glycoprotein subclasses	
			−	No absolute specificity	
		Boronic acid	+	Straightforward enrichment of glycoproteins	[43, 45, 47]
		Mild Hydrazide chemistry	+	Sialic acid specific enrichment of both N- and O-glycoproteins	[80, 99, 122]
			+	Covalent conjugation enables harsh wash procedures	
			−	Non-sialylated structures not enriched	
		SimpleCells (Lectin)	+	Simple and uniform O-glycosylation	[35, 106]
			−	Limited to genetically modified cell lines	
Targeted	Glycopeptides	Antibody	+	High specificity for targeted glycopeptides	[60]
			−	Limited by availability of specific antibodies	
Global	Glycopeptides	Lectin	+	Readily available and cheap	[34, 101–103, 110, 138, 145]
			+	Enrichment of glycopeptide subclasses	
			−	No absolute specificity	
		Hydrazide chemistry	+	Sialic acid specific enrichment of N-glycopeptides	[97, 100]
			+	Covalent conjugation enables harsh wash procedures	
			−	Applicability not demonstrated for O-glycopeptides	
		TiO_2_	+	Specific for sialic acids and phosphates	[115–117]
			+	Enrichment of both N- and O-glycopeptides	
			−	Non-sialylated structures not enriched	
		HILIC	+	Rapid and simple enrichment of glycopeptides	[40, 55, 64, 65, 136]
			−	Limited to hydrophilic glycopeptides	
		Boronic acid	+	Straightforward enrichment of glycopeptides	[46, 48]
			−	Not demonstrated for biological mixtures	
LC-ESI-MS/MS-methods					
	Glycopeptides	CID	+	High sensitivity and speed	[56, 57, 59, 62, 63, 73, 75, 80, 99, 142]
			+	MSn capability	
			+/−	Predominantly glycosidic fragmentation	
		HCD	+	High sensitivity and speed	[35, 61, 146]
			+	Both glycosidic and peptide fragmentation	
			+	High resolution MS2-spectra	
			−	Limited to Orbitrap instruments	
		ECD, ETD	+	Peptide fragmentation with glycans intact	[35, 63, 66, 68, 99, 146–148]
			−	Low sensitivity	
			−	Limited to low m/z precursor ions	

### Lectin purification

Two distinct classes of CBPs are the lectins and the glycan binding antibodies. (Another class of CBP is the glycosaminoglycan binding proteins, which will not be addressed here). Lectins were first discovered in plants more than 100 years ago (for details see the review by Sharon and Lis [21]) but have now been described in all living organisms and play significant roles in many biological systems (chapter 46 of reference [1]). The term “lectin” (lek’tin) is derived from the Latin word legere, meaning: pick out or choose, and is now the generally accepted term for this type of CBPs. However, some lectins still bear the older designation ‘agglutinin’ in their commonly used abbreviations. This is due to the classical method of detecting lectins by which human or animal red blood cells were tested for agglutination. To determine that the agglutinating agent is a true lectin it must be possible to inhibit the agglutination by carbohydrates. Lectins do not display any enzymatic properties and may bind both to free oligosaccharides and to carbohydrate moieties on glycoproteins and glycolipids. Although all lectins by definition are primarily carbohydrate-binding proteins, many plant lectins show additional binding specificities not directed towards carbohydrate residues [22, 23]. Such binding specificities may be part of the explanation for “the lectin riddle”, *i.e.* the finding that glycoproteins of complex biological samples have essentially the same glycan structures irrespective of their retention on lectin affinity columns or not [24]. Thus, it can not be expected that intact glycoproteins and protease digested glycopeptides originating from the same glycoprotein will have the same affinity to a specific lectin. Typically single-site binding affinity of many lectins appears to be low (K_d_ in the micromolar range) although some lectins show higher affinity with K_d_ in the nanomolar range [25]. The low affinity lectins require multivalent binding to glycans to obtain high avidity *in vivo* [1, 26]. The advantage of using lectins is that they are rather cheap and together they provide a broad panel of glycan binding proteins, which may be used in affinity chromatography, lectin blotting and affinity electrophoresis such that some glycoproteins can be separated and/or characterized based on glycan structures and protein glycoforms [27].

Concanavalin A (ConA) is the most extensively used lectin, which shows specific binding towards α-linked mannose/glucose (Man/Glc) typically found on N-linked glycans [28]. The second most common lectin used for glycoprotein characterization and/or enrichment purposes is the wheat germ agglutinin (WGA) that show binding towards *N*-acetylglucosamine (GlcNAc) [29] but also to *N*-acetylneuraminic acid (Neu5Ac) residues [30]. Other commonly used plant lectins, that specifically bind to Neu5Ac terminated oligosaccharides with subterminal galactose (Gal) or *N*-acetylgalactosamine (GalNAc) residues, are the *Sambucus nigra* (elderberry) lectin (SNA, ELB) that recognizes Neu5Acα2-6Gal and Neu5Acα2-6GalNAc structures [31], and *Maackia amurensis* leukoagglutinin (MAL), and *Maackia amurensis* hemagglutinin (MAH) from *M. amurensis* seeds [32], which recognize Neu5Acα2-3Galβ structures [33].

Although lectin affinity chromatography has mostly been used for isolation of unique glycoproteins two global strategies, for enriching a range of glycoproteins/glycopeptides sharing a common glycan structure, have recently been introduced using Jacalin and *Vicia villosa* lectins which specifically recognize the GalNAcα1-O- residues typical for mucin type glycans O-linked to polypeptide sequences [34, 35], see below.

### Immunopurification

Immunopurification or immunoprecipitation (IP) refers to the method of using specific antibodies directed to specific antigens (epitopes) to target and purify single proteins. Glycan binding antibodies or protein binding antibodies may both be used for purification of specific glycoproteins (targeted immunopurification). As for lectins, the antibodies may be immobilized to carrier matrices such as agarose or magnetic beads [36]. The antibody-coated beads are mixed with the protein sample and the targeted proteins are interacting with the antibody and captured onto the beads and thus become immunoprecipitated. Alternatively, the antibody and sample may first be mixed and protein A/G coated beads are then added to specifically bind the antigen-antibody complex from the mixture. The most common approach has been to use the highly-porous agarose beads (agarose resins or slurries) as they tend to have high binding capacity, where almost all possible binding sites on the agarose particle (50–150 μm in size) are available for binding antibodies. Magnetic beads on the other hand, lack a porous center to increase the binding capacity, but the magnetic beads are significantly smaller (1–4 μm), and offer a more effective surface area-to-volume ratio for maximal antibody binding [36]. In some cases, there is no antibody available to target a protein and therefore recombinant tagging technologies are used. The disadvantage of this technique is that glycosylation is in most cases cell and species specific and thus recombinantly expressed glycoproteins are often glycosylated differently compared to the native proteins. The advantage of the recombinant technique is the ability to introduce a selected targeted epitope for immuno- or affinity purification such as His-, Flag-, c-Myc, GFP-, V5-tags, for which commercial purification kits are available. A recent review nicely covers the topic on protein depletion, pre-fractionation and enrichment of proteins for proteomics [37].

## Hydrophilic interaction chromatography and boronic acid purification of glycoproteins and glycopeptides

Hydrophilic interaction chromatography is based on normal phase chromatography, which uses a hydrophilic stationary phase *e.g.* silica, sepharose or cellulose, for retaining glycopeptides owing to the hydrophilicity of the glycan part [38, 39]. HILIC can thus be used to separate protease-digested glycopeptides from other peptides. Often pronase, which is a mixture of proteases from *Streptomyces griseus*, which can cleave almost any peptide bond and thus produce rather short peptide fragments, has been used prior to the HILIC step. Mostly pronase digested N-glycopeptides, carrying relatively larger glycans compared to O-glycans, has been studied, but there are also some examples of O-glycopeptide studies using HILIC [40, 41]. Miniaturized protocols using HILIC microtips packed with cotton wool has been developed for simple and rapid enrichment of tryptic N-glycopeptides [42].

The use of boronic acid functionalized solid supports has also been introduced for the purification of glycopeptides and glycoproteins [43–48]. The retention to the solid support is based on binding between *e.g.* phenyl boronic acid derivatives and the cis-diols of glycans at pH > 8 and then release of the glycans by acidification. The characteristic binding specificities of the HILIC and boronic acid approaches, where the former is based on weak hydrophilic interaction and the latter on covalent binding, make these methods particularly useful for global approaches within the fields of glycoproteomics/glycopeptidomics.

## Targeted O-glycoproteomics; structural characterization of single glycoproteins using LC-MS/MS

**Fig. 1:**
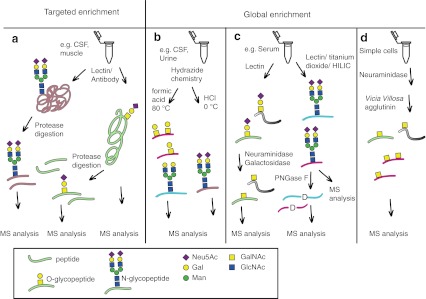
Schematic view of glycoproteomics methods for targeted and global enrichment of glycoproteins and glycopeptides. **a** Typical affinity enrichment of targeted glycoproteins from biological samples. The glycans may be N-linked, O-linked or both. Enriched glycoproteins (as well as endogenous glycopeptides) may be subjected directly to MS analysis, or may be digested by proteases before the MS analysis of glycopeptides and peptides. **b** Enrichment through mild periodate oxidation and hydrazide chemistry for sialic acid specific isolation of both N- and O-glycopeptides. The sialic acids are hydrolyzed by formic acid (HCOOH) treatment at 80 **°**C for both N- and O-glycopeptides, but retained after cold HCl treatment. Enrichment of O-glycopeptides *via* HCl release has not yet been demonstrated. **c** Lectin based strategies for the global enrichment of *e.g.* sialylated core 1 O-glycopeptides (*left arrow*) or N-glycopeptides (*right arrow*). Glycosidases may be used to trim down the O-glycans and PNGase F may be used to remove N-glycans from N-glycopeptides and glycoproteins. Enzymatically modified or intact glycopeptides are then analyzed by MS. **d** The Simple cell global approach produces truncated glycoforms of O-glycoproteins, which after enzymatic treatment allows for the isolation of simplified O-glycopeptides finally enriched by *VVA* lectin chromatography and analyzed by MS

In order to efficiently characterize site-specific O-glycan structures of O-glycoproteins several strategies, as exemplified above, must often be used to achieve sufficient purification of the targeted O-glycoproteins and/or O-glycopeptides (Fig. 1a). Historically, O-glycopeptides originating from protease digested O-glycoproteins were purified by the use of reversed phase HPLC and the peptide sequence determined by Edman degradation [49–53] or sometimes amino acid composition analysis [54]. The inability to detect the Edman product for a specific Ser or Thr was used to indicate that this residue was a true glycosylation site. Large amounts of pure glycoprotein are however needed for Edman degradation. Due to higher analytical sensitivity, high mass resolution and high speed, mass spectrometric methods have become dominating in the field of proteomics and, as we present in this review, of glycoproteomics.

### Glycopeptide analysis

The online coupling of nano liquid chromatography (LC) with electrospray ionization (ESI) and tandem mass spectrometry (MS/MS) makes it straightforward to purify peptides and glycopeptides and perform the mass measurements as the glycopeptides elute from the column. Most often C18 reversed phase chromatography is used, but also hydrophilic interaction liquid chromatography (HILIC) has been used for glycopeptide purification purposes [40, 55]. In the following paragraphs analytical characteristics of various fragmentation strategies for tandem mass spectrometry of glycopeptides are being outlined.

### CID and HCD MS fragmentation strategies

**Fig. 2:**
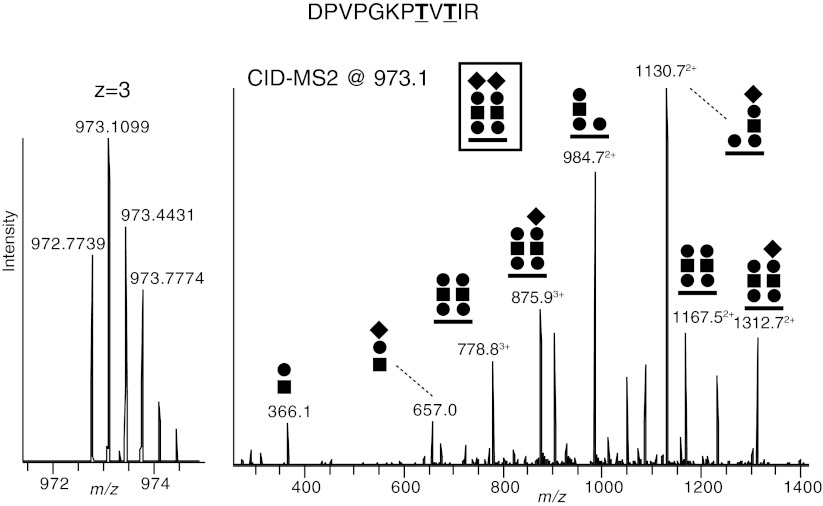
Typical CID fragment ions obtained from LC-MS/MS of tryptic glycopeptides. An expansion of a precursor MS1 spectrum for a tryptic glycopeptide from human α-dystroglycan (DPVPGKPTVTIR) is shown with the high resolving power of FT-ICR instrumentation (*left*) and the resulting CID-MS2 spectrum (*right*). The deduced glycan structure (*boxed*) and proposed structures of selected glycopeptide and glycan fragments are annotated. The composition of this glycopeptide was deduced from the stepwise loss of saccharide units from the glycopeptide and based on the simultaneous presence of analogous glycopeptides lacking one or two Neu5Ac saccharides, identified in simpler CID spectra [57]. ETD fragmentation was recently used to provide peptide backbone fragmentation between the two Thr residues showing the presence of two separate Man-*O*- glycans [77]. The monoisotopic mass of the precursor (*m/z* 972.7739, *z* = 3) is 3.2 ppm off from the theoretical value. Black circle, Hex (Gal and Man); black square, HexNAc (GlcNAc); bold line, peptide. Modified from [57]

**Fig. 3:**
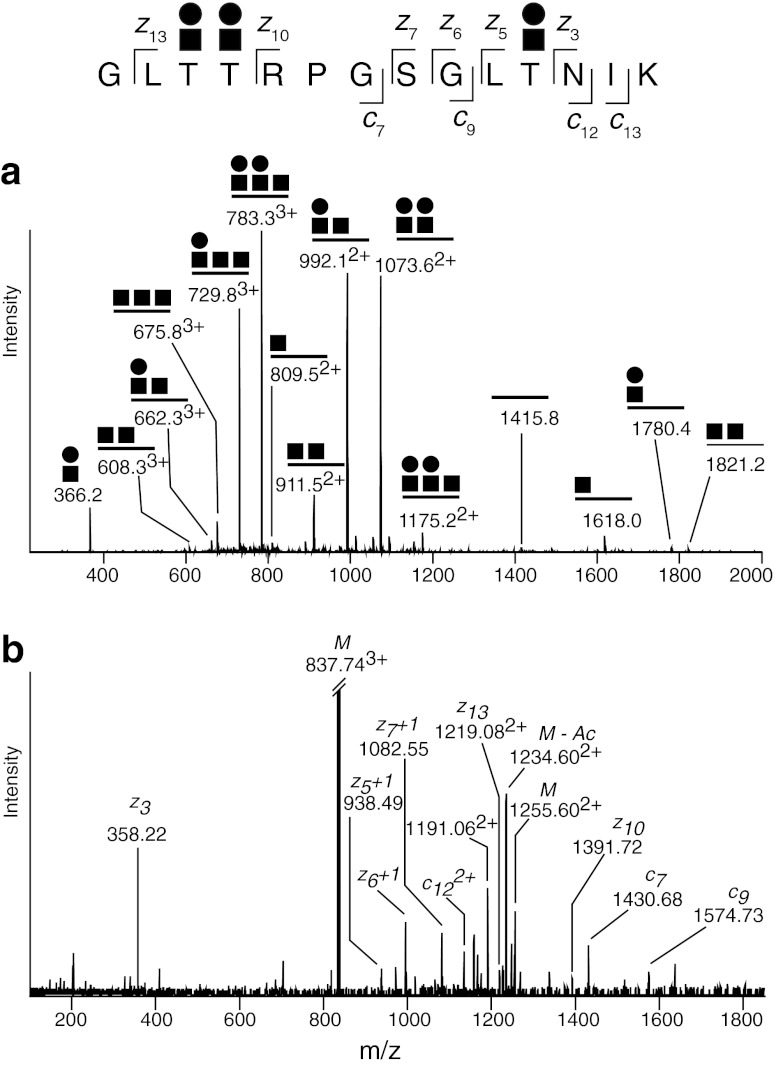
Typical CID and ECD fragment ions obtained from LC-MS/MS after tryptic digestion of human amyloid precursor protein. **a** CID-MS2 fragmentation of the tryptic glycopeptide GLTTRPGSGLTNIK modified with three separate HexHexNAc-O-Thr glycans. Prominent glycosidic fragmentations are observed, which allows the glycan sequences to be verified. The lack of (HexNAc)_2_ (*m/z* 407) and Hex(HexNAc)_2_ (*m/z* 569) oxonium ions in the CID-MS2 spectrum indicates that the peptide is modified with three separate HexHexNAc structures as opposed to *e.g.* one core 2 and one core 1 glycan (**b**) ECD spectrum of the same precursor ion as in panel **a**. The O-glycopeptide is fragmented into c- and z-type ions, without disrupting the labile HexHexNAc-O-Thr modifications, thereby allowing the attachment sites to be determined. Modified from [60]

Starting from a mixture of protease digested peptides and O-glycopeptides, obtained by any of the affinity based glycoprotein purification strategies described above and in Fig. 1, a number of recent studies have appeared where tandem MS techniques for the site-specific characterization of protein O-glycan structures have been used [56–72]. An important limitation related to O-glycopeptide analysis using tandem MS is that collision-induced dissociation (CID), the most frequently used fragmentation technique in LC-ESI-MS/MS, essentially results only in glycosidic fragmentation of the glycan leaving the peptide intact (Figs. 2 and 3). Thus, only the saccharide composition and the sequence of the glycan part may be determined by analysis of a CID-MS2 spectrum. Glycan oxonium ions *e.g.* Neu5Ac^+^ (292 atomic mass units (amu) and its loss of water to 274 amu, HexHexNAc^+^ (366 amu), (Hex)_2_HexNAc^+^ (528 amu) and Neu5AcHexHexNAc^+^ (657 amu) are however diagnostic ions [73] (Fig. 2), which give important information regarding the saccharides present. Nevertheless, when using positive ion mode detection of fragment ions it is not possible to tell whether *e.g.* a HexNAc is a GalNAc or a GlcNAc, the absolute glycan identification must be determined by other means, or be tentatively assigned from known glycosylation pathways related to the glycoproteins studied.

After protease digestion of the targeted O-glycoprotein the LC-MS/MS analysis will also detect peptides originating from the O-glycoprotein (Fig. 1a). The identities of these peptides are obtained by searching protein databases for the precursor ion mass and by identification of fragment ions containing the peptide backbone in the CID-MS2 spectrum. The identities of the O-glycopeptides may be established by using the Glycomod tool [74], which attempts to match an appropriate glycan composition to all possible tryptic peptides containing serine and/or threonine to add up to the measured precursor mass. A higher accuracy of the measured precursor, *e.g.* by using high-resolution instruments such as a FT-ICR or Orbitrap, results in fewer but more reliable hits. Proposed glycan compositions, which match that of previously characterized glycans are complemented with an HTML link to the GlycosuiteDB, where further information and relevant examples from the literature can be accessed [74]. Glycopeptide hits are then verified or dismissed by inspection of the CID-MS2 spectra so that the glycopeptide proposed by Glycomod matches the assigned CID-MS2 spectrum (Fig. 2) [57]. Also, further fragmentation (CID-MS3) of glycopeptide fragments may be helpful in the determination of glycopeptide structure, especially if the O-glycan structure is complex. Occasionally, the absolute identification of O-glycopeptides can be obtained by the presence of peptide backbone fragmentation even during CID conditions [57, 59].

LC-MS/MS using CID fragmentation of tryptic glycopeptides has recently been used in four studies of α-dystroglycan (α-DG) [56–58, 75] (Fig. 2). Aberrant glycosylation of α-DG is implicated in a number of congenital muscle dystrophies, which makes this protein an important target for glycosylation analyses. An unusual O-glycan based on O-Man linkage to the protein, which is important for the α-DG function, has been described [76]. Yoshida-Moriguchi *et al.* recombinantly expressed the mucin region of α-DG in HEK293 cells and used *Wisteria Floribunda* agglutinin (WFA) lectin, which binds terminal GalNAc residues linked to the 3′ or 6′ position of Gal, for the affinity purification of GalNAc modified α-DG. After tryptic digestion they used ESI-LC-MS/MS for the site specific characterization of α-DG glycopeptides, and NMR analysis of released saccharides for determining their anomeric and linkage specific composition, and found a novel GalNAcβ1-3GlcNAcβ1-4Man-*O*- structure, which was substituted with a 6-*O*-phosphate group at the Man residue [75]. A recent study applying similar strategies using recombinant mouse α-DG expressed in HEK293 cells did also identify this novel O-mannosylated structure but without the modification of the phosphorylated Man [58]. In this study, the secreted α-DG fusion protein was affinity purified using protein G, digested with trypsin and the glycopeptides were further enriched using WFA lectin chromatography.

Two studies have reported the characterization of glycosylation pattern of α-DG from native skeletal muscle in rabbit [56] and in human skeletal muscle [57]. The O-glycosylation core structures and site occupancies were determined and showed similar results regarding the GalNAc-*O*- and Man-*O*- core structures but differences were observed for the site occupancy of the Man-*O*- structures between the two studies. Lectin affinity chromatography using wheat germ agglutinin (WGA) was used both before laminin-sepharose affinity purification of the rabbit α-DG, and before immunopurification of the human skeletal muscle α-DG with the α-DG specific *VIA*-4 antibody. Different glycoforms of native human and rabbit skeletal muscle α-DG may thus have been purified and explain the glycosylation discrepancies [77].

Higher energy collision dissociation (HCD) is a recently introduced fragmentation technique of Orbitrap instrumentations [78] where O-glycans are readily expelled from the O-glycopeptide, also providing peptide backbone fragmentation useful for identification purposes [61]. As a rule of thumb it is more likely that, when using CID or HCD, peptide backbone fragmentations will be observed for O-glycopeptides carrying more simple O-glycans. Strategies for removing parts of the O-glycans have thus been introduced (Fig. 1c), *e.g.* using glycosidases, to increase the chances of peptide fragmentation in the presence of partially intact glycosylations, which thus can be used for pinpointing the O-glycosylation site(s) in glycopeptides containing several Ser or Thr residues [79, 80]. Such strategies have specifically been used in global O-glycoproteomics studies and are discussed below.

The CID fragmentation analysis of O-glycopeptides may also be complemented with O-glycosylation site tagging methods in order to pinpoint the glycosylation sites. Lee *et al.* [62] isolated Apolipoprotein E (ApoE) from human blood-derived macrophages using immunopurification. Then, they prepared ApoE peptides and glycopeptides using in-gel digestion, and identified a range of O-glycopeptides including the known Thr212 glycosylation site but also novel glycopeptides from the C-terminal tryptic peptide 301-VQAAVGTSAAPVPSDNH-317. To pinpoint the glycosylation sites of this glycopeptide they performed β-elimination using methylamine vapor in order to specifically tag the glycopeptide at the site of glycosylation [81, 82]. This covalent modification was not fragmented during CID conditions and the glycosylation site could thus be selectively pinpointed to Ser308 (Ser290 excluding the signal peptide) using analysis of the peptide backbone *b*- and *z*-ion fragments [62].

Chemical β-elimination (mild alkaline treatment) has traditionally been used to release O-linked oligosaccharides from glycoproteins. Unfortunately, this strategy is not well suited for glycoproteomic studies. Conventional β-elimination (reductive or non-reductive) results neither in the complete conversion into deglycosylated species nor does it preserve the structural integrity of the peptide backbone [83]. However, alternative strategies have been developed to circumvent the destructive conditions of conventional beta elimination. Wells *et al.* developed a protocol for beta elimination followed by Michael addition with dithiothreitol (BEMAD) specifically designed for glycoproteomic studies [84]. The procedure is milder and works relatively well for releasing GlcNAc-*O*- at Ser and Thr residues without degrading the peptide backbone. The BEMAD procedure also adds a uniform mass tag (dithiothreitol, DTT) to the formerly O-glycosylated amino acid, thereby simplifying the identification of the modified site. However, the mild BEMAD procedure does not readily release O-linked GalNAc residues [85].

### ECD and ETD MS fragmentation strategies

The ECD and ETD [86] fragmentation methods may be used as a complement to CID for the characterization of glycopeptides and may be performed subsequent to each other on the same precursor ions during LC-MS/MS runs. ECD and ETD are known to provide peptide backbone fragmentation into *c*- and *z*-ions, also in the presence of labile post-translational modifications such as glycosylation (Fig. 3) [87, 88]. Thus, these techniques provide direct peptide fragmentation of glycopeptides useful for identification purposes and for pinpointing glycan attachment sites. However, the ECD and ETD fragmentation methods are less sensitive compared to CID and HCD and the *m/z* values of precursor ions should be below 1,000 amu in order to obtain efficient peptide fragmentation. Perdivara *et al.* used ETD for the characterization of O-glycopeptides derived from trypsin and chymotrypsin cleavage of recombinant human amyloid precursor protein (APP) expressed in Chinese hamster ovary (CHO) cells [63]. APP is known to be both N- and O-glycosylated [89–91], but no O-glycosylation sites had previously been defined. The site-specific characterization of O-glycosylation sites was accomplished with LC-MS/MS using a combination of CID and ETD, and the sites were pinpointed to Thr291, Thr292 and Thr576 of the APP695 isoform [63].

### MS analysis of endogenous glycopeptides and small glycoproteins

A series of peptidomics studies have appeared where endogenous O-glycopeptides have been identified. Zougman *et al.* used CSF samples and separated endogenous peptides from proteins using 10 kDa molecular cut-off ultracentrifugation membranes [61]. The peptide fraction was then analyzed with ESI-LC-MS/MS using CID and HCD fragmentation. Due to peptide fragmentation of O-glycopeptides using HCD-MS2 a few novel glycopeptides and O-glycosylation sites could be defined from *e.g.* heparin-binding EGF-like growth factor and insulin-like growth factor-2. Balog *et al.* used HILIC chromatography to purify endogenous glycopeptides in the urine of individuals infected with the *Schistosoma mansoni* parasite [64]. They used matrix assisted laser desorption and ionization-time of flight (MALDI-TOF) MS/MS and ESI LC-MS/MS and found endogenous glycopeptides typically containing a triply fucosylated glycan with core 2 like structure. These fucosylated structures were absent in uninfected individuals. By fragmentation of the peptide backbone they identified the peptide as 85-WDLDPEVRPTSAVAA-99 from Apo-CIII, thus demonstrating a change in host protein glycosylation in response to the parasite infection. Recently, Pacchiarotta *et al.* purified endogenous Fibrinogen alpha chain O-glycopeptides from urine samples and found that the glycopeptide level was raised for individuals with urinary tract infection [65].

Halim *et al.* used an immunopurification MS protocol [92–94] to enrich endogenous amyloid-β glycopeptides originating from the amyloid precursor protein (APP) in human cerebrospinal fluid (CSF) samples. A series of relatively large APP/Amyloid β (Αβ) glycopeptides, spanning from *e.g.* residue −57 to residue 15 (numbered in relation to Asp1 of the Aβ sequence) and with 1 to 4 (Neu5Ac)_2_HexHexNAc glycans in the sequence, were identified using CID [60]. Verification of the identities was obtained by diagnostic peptide backbone fragmentations in the presence of O-glycosylations, and by the co- presence of the corresponding unglycosylated peptides in the LC-MS/MS runs, which also provided semi-quantitative information on the abundance of glycosylation. In the same LC-MS/MS runs using CID also shorter Aβ1-Χ glycopeptides, *e.g.* Aβ1-15 (DAEFRHDSGYEVHHQ), were identified having a Neu5AcHex(Neu5Ac)HexNAc-*O*- glycan attached to the peptide. ECD fragmentation showed that the glycan was uniquely attached to Tyr10, defining for the first time a tyrosine glycosylation with sialic acid containing glycans. Steentoft *et al.* recently confirmed the possibility of tyrosine glycosylation using a Simple cell methodology [35], see below.

Even intact glycoproteins may be studied with ECD/ETD fragmentation. Apolipoprotein CIII (Apo-CIII) is a small O-glycoprotein (79 residues), which is known to be O-glycosylated at Thr94 [95]. Ito *et al.* analyzed by mass spectrometry the entire protein and found the glycan to be composed of sialylated and disialylated core 1 structures [96]. Mazur *et al.* used *top-down* ETD fragmentation analysis of the intact Apo-CIII O-glycoprotein [66] and pinpointed the glycosylation sites of the Neu5AcHexHexNAc, (Neu5Ac)2HexHexNAc O-glycans O-glycans to either one of the three most C-terminal Ser/Thr residues. Direct pinpointing of the glycosylation site to Thr94 in the tryptic peptide 79-DKFSEFWDLDPEVRPTSAVAA-99 was accomplished by Ueda *et al.* [67] using MALDI-TOF/TOF analysis.

## Global O-glycoproteomics; enrichment and structural characterization of O-glycopeptides

The global mapping of O-glycosylation sites of proteins in complex mixtures is becoming increasingly available through the analysis of O-glycopeptides obtained from protease digestion of such protein samples. However, to accomplish efficient O-glycoproteomics from biological samples it is necessary to selectively enrich the O-glycopeptides from the vast amount of unglycosylated peptides (Fig. 1b–d). Secondly, various mass fragmentation techniques are needed to characterize the possible Ser/Thr and Tyr attachment sites, and ultimately to characterize the glycan heterogeneities of such glycoproteins. The following paragraphs will give examples of such strategies.

### Sialic acid capture-and-release strategy

**Fig. 4:**
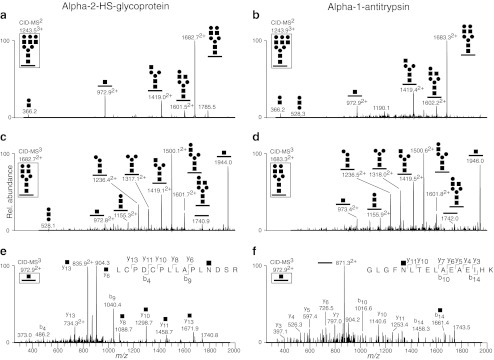
FTICR-MS/CID-MS^n^ analysis of N-glycopeptides enriched from bovine serum. **a** CID-MS2 of the triantennary N-glycopeptide LCPDCPLLAPLNDSR of bovine alpha-2-HS-glycoprotein. **b** CID-MS2 of the triantennary N-glycopeptide GLGFNLTELAEAEIHK of bovine alpha-1-antitrypsin. **c** and **d** show CID-MS3 of the most abundant fragment ion in (**a**) and (**b**), respectively. **e** and **f** show CID-MS3 of the Y_1_-type (*m/z* 972.9) fragment in (**a**) and (**b**). The CID-MS3 spectra of the most abundant fragment ions at *m/z* 1682.7 (**a**) and *m/z* 1683.3 (**b**) were used to verify the N-glycan structure. The N-glycopeptide identities were verified only when the fragment ions at *m/z* 972.9 were analyzed by CID-MS3. The structure of precursor ions subjected to CID-MS^n^ fragmentation are shown boxed in each panel

Nilsson *et al.* introduced a hydrazide chemistry method for the sialic acid capture-and-release of both N- and O-glycopeptides (Fig. 1b) [80], which is based on two well established facts regarding sialic acid containing glycans: i) sialic acids may be selectively oxidized on the glycerol chain by using mild periodate oxidation and ii) sialic acid glycosidic bonds may be selectively hydrolyzed by mild acid treatment. Through this approach it was possible to capture sialylated glycoproteins onto hydrazide beads, wash extensively, perform trypsin digestion and then release desialylated N- and O-glycopeptides with 0.1 M formic acid at 80 °C for 60 min. Also, the Nishimura group presented a similar methodology named reversed glycoblotting [97], see below. Nano LC-ESI-MS/MS on a hybrid Fourier transform ion-cyclotron resonance (FT-ICR) spectrometer (LTQ-FT) was used with the precursor ions measured with high accuracy in the FT-ICR detector and then selected, fragmented with collision induced dissociation (CID) and measured in the linear ion trap quadrupole (LTQ). The strategy was validated on model proteins, such as human serum transferrin and bovine fetuin, and was then initially used for the characterization of glycoproteins and glycopeptides in human cerebrospinal fluid samples. The CID-MS2 spectra of O-glycopeptides typically showed loss of Hex into the peptide + HexNAc ion and loss of HexHexNAc into the peptide ion. The O-glycans were thus composed of HexHexNAc-O- in support of a core 1 (Galβ3GalNAcα1-*O*-Ser/Thr) structure. Furthermore, CID-MS3 of the peptide ion gave peptide backbone fragmentation into *b*- and *y*-ions, which was used to identify the peptide by Mascot protein database searches (see below for the analogous CID-MS2/MS3 strategy regarding N-glycopeptides (Fig. 4)). The precursor mass for the Mascot search was set to the measured glycopeptide mass minus the calculated mass of a HexHexNAc ion (365.1322 amu) in order to take advantage of the high mass accuracy of the precursor, measured in the FT-ICR cell. With this approach 44 O-glycosylation sites on human CSF proteins were identified [80].

Unfortunately, this CID-MS2/MS3 approach does not generally give the Ser/Thr glycan attachment sites of O-glycopeptides containing several Ser/Thr since the glycan is lost before the peptide backbone is fragmented. However, occasionally peptide fragmentation took place in the presence of an intact HexNAc-O-. This was especially true for proline containing peptides giving rise to prominent *b*- and/or *y*-ion peaks resulting from fragmentation at the N-terminal side of proline [98], which thus could be used to pinpoint the exact attachment sites, or at least exclude some of them. By using a combination of CID and ECD fragmentation (Fig. 3) three glycosylation sites of the Amyloid Precursor Protein (APP770 isoform) were characterized [60], one of which was identical to the Thr576 (APP695 isoform) identified by Perdivara *et al.* [63].

Recently, Halim *et al.* used this sialic acid capture-and-release strategy on human urine samples [99] and introduced the systematic use of ECD for pinpointing both the N- and the O-glycosylation sites of urinary proteins. In total, 58 N- and 63 O-linked glycopeptides from 53 glycoproteins were characterized and the combined use of CID and ETD allowed for the exact identification of Ser/Thr attachment site(s) for 40 of 57 O-glycosylation sites, 29 of which were not reported before [99]. The core 1 like structure (HexHexNAc-O-) was the major glycoform found for all O-glycopeptides but also a core 2 like structure (Hex(HexHexNAc)HexNAc-O- was identified for a few of the O-glycopeptides, demonstrating the possibility to perform a global characterization of not only O-glycosylation sites but also that of glycan structures. One advantage of using a chemistry based method such as a covalent capture using hydrazide chemistry is that the solid phase can be extensively washed in order to get rid of virtually all background binding of unglycosylated peptides. However, upon treatment with formic acid, even under mild conditions peptide bonds after Asp residues are occasionally hydrolyzed and sialic acids are always lost. An elegant way to avoid hydrolyzing the sialic acid glycosidic bond is to use cold 1 M HCl as was recently shown by Kuroguchi *et al.* [100].

### Lectin based strategy

Darula *et al.* presented a lectin-based approach for the global enrichment of O-glycopeptides originating from bovine serum proteins (Fig. 1c) [34]. After tryptic digestion of the serum sample they used Jacalin-coated columns, which recognize terminal GalNAcα1-O- and GalNAcα1-O- substituted at the 3-OH position, for the affinity enrichment of O-glycopeptides. The major glycoforms isolated carried Neu5AcGalβ3GalNAc-O- glycans and mainly ETD was used for glycopeptide identifications. The authors pinpointed 26 glycosylation sites from bovine serum glycoproteins, and later expanded the list with about 10 additional ones [101, 102]. Very recently Darula *et al.* introduced the use of complementary chromatographic steps, *e.g.* ion exchange chromatography, to further fractionate bovine serum samples both at the glycoprotein and the glycopeptide levels. With this approach a total of 124 O-glycosylation sites from 51 glycoproteins were identified [103]. The authors found that the ionization efficiency was low when the *m/z* values approached 1,000 and that it was advantageous to remove both the Neu5Ac and Gal residues by using neuraminidase and β-galactosidase to reduce the *m/z* values of the glycopeptides by 291 and 162 Da, respectively. This procedure improved the ETD ionization efficiencies and the remaining GalNAcα1-O- residues were left intact for the identification of the exact glycosylation sites. One example of an O-glycosylation presented was Ser296 of bovine fetuin, which independently was found also by Halim *et al.* [80] and by Zauner *et al.* [40]. Zauner *et al.* used proteinase-K digestion in combination with hydrophilic interaction liquid chromatography (HILIC) to purify both N- and O-glycopeptides from bovine fetuin in their study.

### The Simple Cell strategy

Steentoft *et al.* recently designed a completely novel strategy to specifically produce only GalNAcα1-*O*- modified O-glycoproteins from cell cultures (Fig. 1d) [35]. They used the zinc finger nuclease gene targeting method [104] to silence the expression of the *cosmc* chaperone, which is essential for the production of functional Gal-T1 transferase [105]. Thus, Gal elongation of the initial GalNAc-*O*- modifications becomes completely blocked. After trypsin digestion of cell extracts they used *Vicia villosa* agglutinin (VVA) affinity columns for the selective enrichment of such truncated GalNAcα1-*O*- substituted O-glycopeptides. For the MS analysis, Steentoft *et al.* used HCD, which fragmented away the GalNAc residue(s) during the MS2 and provided peptide backbone fragmentation for glycopeptide identification. Also, ETD was used to pinpoint O-glycosylation sites, and as many as 10 glycosylation sites could be mapped on some tryptic peptides. With this approach more than 350 O-glycosylation sites from 100 human proteins have now been identified [35]. Most recently, the SimpleCell strategy was extended to study the isoform-specific functions of ppGalNAc-Ts [106].

The three global glycoproteomics strategies for the characterization of O-glycosylations described above (Fig. 1b–d) have a common feature *i.e.* native O-glycopeptides are structurally simplified during the enrichment procedure. For the sialic acid capture-and-release method sialic acids are removed during the enrichment procedure leaving only the core (mostly core 1) glycopeptides to be analyzed. For the jacalin lectin approach sialylated core 1 substituted O-glycopeptides are trimmed down by the use of α-neuraminidase and β-galactosidase. Finally, for the Simple Cell approach only GalNAcα1-*O*- substituted O-glycopeptides are produced and enriched. These simplifications of glycan structures are beneficial for MS analysis since the fragmentation spectra are much simplified. The reduction of glycan mass and thus also the *m/z* value is also beneficial for the use of ECD and ETD fragmentation techniques. A disadvantage of studying only GalNAc substituted O-glycopeptides is however that all structural information of possible glycan extensions is lost.

For lectin based approaches, and other methods dependent on weak affinity purification, there still remains difficulties in selectively purifying only one glycoprotein or only one glycoform of a protein [24], and often there remains a great deal of unglycosylated peptides present in the LC-MS/MS runs. To minimize this background Steentoft *et al.* used an initial HCD, which produces a major HexNAc^+^ oxonium ion at *m/z* 204 when the precursor is a GalNAc substituted glycopeptide. The presence of the critical *m/z* 204 peak was programmed to trigger a subsequent ETD fragmentation event on the same precursor. HCD is a fast fragmentation method compared to ETD, and the triggering of ETD only for true glycopeptides maximized the number of precursors that could be fragmented [35].

## N-glycoproteomics

N-glycosylated proteins are certainly the most well studied class of glycoproteins. The combination of proteomics and glycomics has proven to be a powerful tool for such studies. However, a comprehensive understanding of N-glycoprotein structure (and function) also requires a detailed knowledge of the site-specific location of N-glycan structures. An important complement to the well-established techniques of proteomics and glycomics is thus glycoproteomics where intact N-glycopeptides, obtained either from targeted single glycoproteins or from global analyses of complex biological mixtures, are studied with respect to both glycan structures, attachment sites and peptide sequences.

Although N-glycoproteins include the high-mannose- (Man_5-9_GlcNAc_2_-*N*-Asn) and hybrid-type (*e.g.* Man_5_(Neu5AcGalGlcNAcMan)ManGlcNAc_2_-*N*-Asn) modifications, secreted and membrane bound glycoproteins are most commonly glycosylated with the sialylated complex-type (*e.g.* Neu5AcGalGlcNAcMan(Neu5AcGalGlcNAcMan)ManGlcNAc_2_-N-Asn) N-glycans (see Fig. 1) [107–109]. Traditionally, researchers have used various pre-analytical strategies to overcome some of the difficulties associated with MS-based characterization of N-glycosylated proteins. Most commonly, N-glycoproteins/peptides are enriched with lectins [110–114], titanium dioxide columns [115, 116], graphite microcolumns- [117], size-exclusion-, hydrophilic interaction liquid chromatography (HILIC) [55, 79, 118], boronic acid- [46] or hydrazide chemistry based approaches (Fig. 1) [119–122]. Many of these protocols typically involve enzymatic hydrolysis of the enriched N-glycoproteins and glycopeptides by peptide:N-glycosidase F (PNGase F), which cleaves the β-aspartyl *N*-acetylglucosaminyl bond of both high-mannose-, hybrid- and complex type N-glycans [123, 124]. The PNGase F treatment thus releases the N-glycans with an intact chitobiose reducing end while hydrolyzing the formerly glycosylated asparagine residue to an aspartic acid. Released N-glycans are subsequently characterized by glycomic techniques while the deglycosylated proteins and peptides are identified by established proteomic methods.

The separation of N-glycans from their protein carriers has its own advantages and disadvantages. In glycomics, sensitive characterization of N-glycan structures is indeed achieved but the site-specific location of these glycans and the identities of their protein carriers are lost. Methods that involve the analyses of N-glycans released from their proteins carriers are not covered here but the readers are instead referred to the following reviews [125, 126].

Hundreds to thousands of N-glycosylation sites of formerly N-glycosylated proteins have now been identified by the proteomic analysis but this approach is still unable to provide any structural information on the actual N-glycans attached to the glycoproteins identified. Not only after PNGase F treatment but also in nature, asparagine can change into aspartic acid and thereby can increase the rate of false positive identifications [127, 128]. However, if PNGase F digestion is performed in H_2_^18^O, the formerly glycosylated asparagine would obtain a +3 mass units shift [129] and thus the N-linked glycosylation site will be specifically labeled by ^18^O and detected by MS. The technique is named isotope-coded glycosylation site-specific tagging (IGOT) [129] and provides direct evidence for N-glycosylation sites.

## Targeted N-glycoproteomics: structural characterization of single N-glycoproteins

In N-glycoproteomics, MS-based methods may be used to elucidate not only glycoprotein identities but also to characterize glycan structures and pinpoint their exact protein attachment sites. This methodology has an absolute requirement for intact glycopeptides. So far, MS-based strategies aimed at analyzing intact N-glycopeptides have generally been limited to the targeted or monoproteic approach, a definition recently used by Dodds *et al.* to describe the glycoproteomic analysis of single glycoproteins [130]. Examples of such studies include MS-analysis of commercially available glycoproteins, *e.g.* apolipoprotein B100, bovine ribonuclease B, asialofetuin and ceruloplasmin [131–134]. Commercially available glycoproteins are typically supplied as highly purified mono-species, which allows a direct analysis of the proteolytic digests to detect and characterize their N-glycopeptides by LC-MS/MS methods. However, researchers have also adopted one of several chromatographic strategies to enhance the sensitivity of their analyses, *i.e.* the use of normal-phase chromatography, HILIC or graphitized carbon to separate N-glycopeptides from non-glycosylated peptides [38, 40, 117, 135–137]. Lectin chromatography has also been used for the enrichment of N-glycopeptides derived from commercially available glycoproteins [110, 138]. In addition, targeted analysis of N-glycoproteins, derived either from biological samples or commercial sources, has been achieved through the analysis of in-gel digested samples following isoelectric focusing (IEF) [139] or 2D-SDS-PAGE separation [140, 141].

## Global N-glycoproteomics: enrichment and characterization of N-glycopeptides

Various enrichment strategies, many of which are already mentioned, have been combined with MS-analysis for the global scale analysis of N-glycoproteins and glycopeptides from biological samples[111, 142]. Hancock’s group used multi-lectin affinity (M-LAC) columns (ConA, WGA and jacalin) to enrich N-glycoproteins from human plasma [143, 144]. Following trypsin digestion of the enriched N-glycoproteins, 25 N-glycopeptides were identified by replicate LC-MS/MS analyses of PNGase A treated and untreated N-glycopeptides. This strategy is based on LC-MS/MS characterization of intact N-glycopeptides and on identification of the peptide sequence by proteomic analysis of the deglycosylated samples. The precursor masses measured by FTICR-MS in the replicate analyses are ultimately used to correlate the fragmentation spectra of specific N-glycopeptides to specifically identify the deglycosylated peptides. Uematsu *et al.* employed a similar approach using ConA to affinity-purify tryptic N-glycopeptides derived from murine dermis and epidermis and by using MALDI-TOF/TOF for the analysis 20 N-glycopeptides carrying high-mannose type N-glycans were identified in PNGase F treated and untreated replicates [145]. The same group also developed “reverse glyco-blotting” for the chemistry-based enrichment of glycopeptides from complex biological mixtures. In their initial approach, oxidized sialic acids of tryptic N-glycopeptides were conjugated to aminooxy-functionalized polymers *via* stable oxime bonds and released by trifluoroacetic acid hydrolysis of the sialic acid glycosidic bonds [97]. Independently, Nilsson *et al.* developed a similar enrichment strategy based on hydrazide chemistry [80]. Using LC-ESI-MS/MS analysis, 36 N-glycopeptides derived from human CSF were characterized with respect to both glycan- and peptide sequences which demonstrated the successful use of a MS-method involving CID-MS2 followed by CID-MS3 of the top-five fragment ions for complete glycan and peptide characterization of N-glycopeptides. This MS-method is dependent on the characteristic CID-fragmentation pattern of N-glycopeptides [73, 142]. Typically, CID-MS2 of N-glycopeptides generates abundant Y_1_-type fragments, *i.e.* peptide + HexNAc fragments, which are further analyzed by CID-MS3 to induce peptide backbone b- and y-type fragmentations. The CID-MS3 spectrum is then used to identify the peptide sequence by database searches, *e.g.* by using the Mascot algorithm. The principle for this MS-method was demonstrated on model N-glycoproteins by Wuhrer and co-workers [38]. In Fig. 4, the applicability of this MS-method is shown for the analysis of two triply charged N-glycopeptides, enriched from bovine serum, with different identities but with the same nominal masses (at *m/z* 1243.5325 and *m/z* 1243.8896). These examples underscore the need for accurate mass measurements of precursor ions and tandem MS fragmentations for confident identification of N-glycopeptides derived from complex biological samples.

In 2010, an improved enrichment strategy for N-glycopeptides, based on hydrazide chemistry, was reported by Nishimura and co-workers [100]. In their refined method, release of sialylated glycopeptides by ice-cold 1 M HCl was reported, along with the identification of 67 N-glycopeptides from mouse serum. Thus, this release strategy represents an alternative method for the enrichment of sialylated N-glycopeptides. More recently, Halim *et al.* explored the N-glycoproteome of human urine using the sialic acid capture-and-release protocol [99], which allowed for the identification of glycan sequence, peptide sequence and glycan microheterogeneity of 58 N-glycopeptides by LC-MS/MS using a combined CID and ECD fragmentation analysis approach. The applicability of HCD and ETD for the sequence analysis of N-glycopeptides has previously been demonstrated [146–148].

## Linking glycans to proteins through data base management

**Fig. 5:**
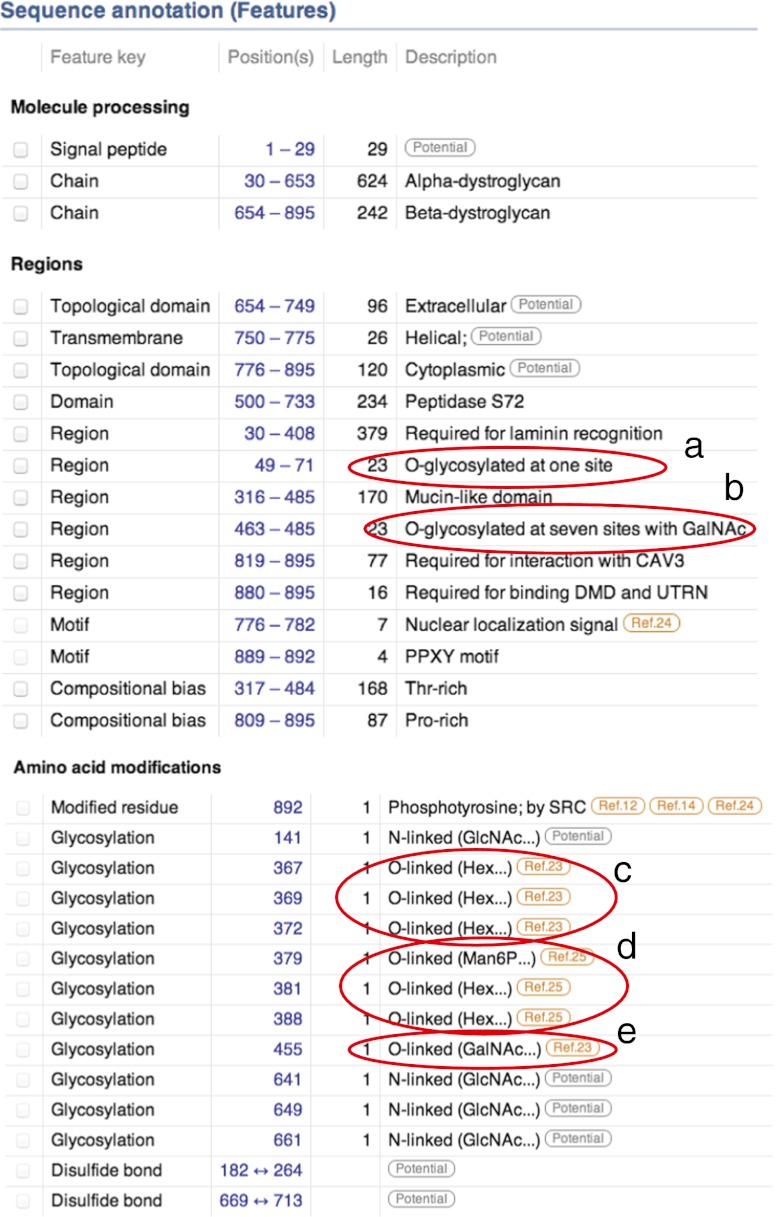
UniprotKB HTML presentation of human Dystroglycan precursor (http://www.uniprot.org/uniprot/Q14118). Specific O-glycosylation features are encircled as **a** reported in [80]; **b** reported in [57]; **c** reported in [57]; **d** reported in [75]; **e** reported in [57]

The complexity of glycans described by glycomics approaches demand unique technologies, unique nomenclature and data handling and should be continuously advanced [149, 150]. However, the novel glycoproteomic approaches, described in this review, are quickly adding new and significant data on the structural modifications of glycoproteins which fit very well also into established protein databases. Thus, although most glycans have not been described in such structural details, as is state-of-the-art in the glyco-science fields, a partial characterization of a glycan or even the mere presence of a glycosylation at any specific amino acid residue in a glycoprotein, is of considerable interest for understanding the role of glycosylation. We have experienced a growing interest from *e.g.* UniProt (Fig. 5), in adding information on glycosylation structure and sites into the Swiss-Prot database, which is now manually being updated on published results. A critical issue is the validity of the actual information, on the glycan structures and their attachment sites (or regions), being reported into the database but as long as references are given manually to the original publications there is no need for either reductionism or overinterpretation of data. When automatic routines become implemented quality assurance must follow to avoid false information to be stored and at that stage a linkage to glycomics databases should probably be established. Hopefully, all groups working with glycoproteomics will from now on systematically submit their data to *e.g.* UniProt to increase the general awareness and availability of novel or confirmatory protein glycosylation sites and their site-specific glycan structures.

## Conclusions

Glycoproteomics has recently evolved as a new and very promising complementary “-omics”. This methodology, designed for a global characterization of complex glycoproteins enriched from cell cultures and from biological samples, gives novel structural information on core glycans and their exact attachment sites on, even low abundant, glycoproteins. Much has been learned from the success of global proteomic analyses both as to preanalytical, analytical and postanalytical strategies, which have thus become applicable also for use in the field of glycoproteomics. However, seemingly minor but very critical alterations in these strategies are necessary for a successful application of the analyses for glycoproteins and glycopeptides. In this review we have described and exemplified some of these critical steps for enrichment of glycoproteins and glycopeptides, for simplification of their glycan structures prior to glycosylation site analysis and for selection of optimal fragmentation techniques and instrumentations for liquid chromatography tandem mass spectrometry. Finally, we have urged the glyco-science community to store novel experimental data on glycan structures and attachment sites in well-established protein databases even when structural information on the complete glycan structures are missing. We have tried to be inclusive as to referring to all valuable contributions in the field but apologize if we have failed and mistakenly left some references out.
